# The role of momentary emotions in promoting error learning orientation among lower secondary school students: An intervention study embedded in a short visual programming course

**DOI:** 10.1111/bjep.12681

**Published:** 2024-03-19

**Authors:** Rahel Schmid, Robbert Smit, Nicolas Robin, Alexander Strahl

**Affiliations:** ^1^ St. Gallen University of Teacher Education St. Gallen Switzerland; ^2^ Paris Lodron University of Salzburg Salzburg Austria

**Keywords:** coding lessons, emotions, error learning orientation, errors, lower secondary education

## Abstract

**Background:**

Students make many errors in visual programming. In order to learn from these, it is important that students regulate their emotions and view errors as learning opportunities.

**Aims:**

This study aimed to explore to what extent momentary emotions, specifically enjoyment, anxiety and boredom, as well as the error learning orientation of students, interacted during a 1‐day course on visual programming in an out‐of‐school learning environment.

**Samples:**

The sample consisted of 269 lower secondary school students (grades 7–9).

**Methods:**

The data were collected in an intervention study, with questionnaires applied directly before and after the course, and with four measurements of state emotions during the course.

**Results:**

The results showed that error learning orientation had an expected effect on the students' emotions at the beginning of the course. The emotions changed positively over the course of the workshop, while the error learning orientation remained stable. No differences in error learning orientation were found between the control and intervention groups. An expected, reciprocal effect of students' emotions on their error learning orientation at the end of the course day could not be found.

**Conclusion:**

Changes in error learning orientation are difficult to achieve during 1‐day courses. Nevertheless, through targeted, pedagogical approaches, which aim to minimize the influence of unfavourable emotions that occur in problem‐oriented learning situations, teachers could help students develop a positive error learning orientation in the long term, whereby errors are viewed as an opportunity for learning.

## INTRODUCTION

A large number of different studies and research findings over the last decade show that the correct handling of errors can significantly foster students' learning (for reviews, see Mera et al., 2022; Metcalfe, 2017). The students themselves have an important role to play in dealing with errors, as the way in which they deal with these is decisive in determining whether the error promotes or hinders learning (Kreutzmann et al., 2014). This depends whether or not students view errors as learning opportunities (Zhang & Fiorella, 2023). A corresponding person's mindset is referred to as error learning orientation (Bligha et al., 2018).

**FIGURE 1:**
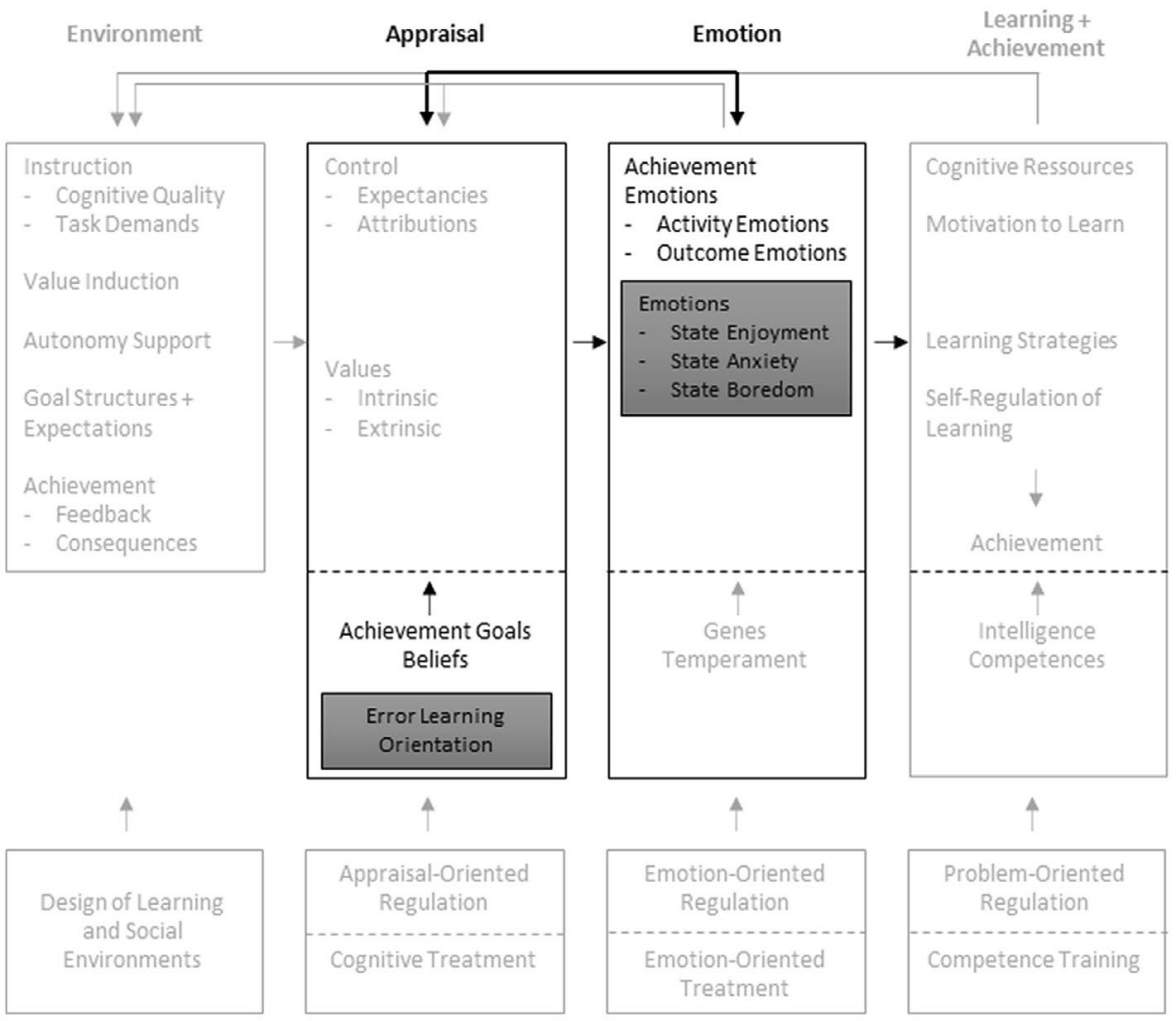
The ‘CVTAE’ adapted from Pekrun et al. (2006). Variables located: investigated variables in grey boxes and investigated paths in black.

When students make errors, they often feel ashamed or unintelligent (Tulis & Ainley, 2011). Several studies indicate that the learning orientation towards errors is linked to emotions (e.g., Tulis & Ainley, 2011; Zhang & Fiorella, 2023). Students who find mistakes embarrassing and wish to avoid them at all costs often feel anxious in performance situations (Pekrun et al., 2006). However, as has been known for some time, not only negative emotions can be felt when making errors but also positive emotions (Diener & Dweck, 1980; Tulis & Ainley, 2011). For example, students who experience mistakes as an incentive often feel proud when they have found the solution (Tulis & Ainley, 2011). In Pekrun's (2006) control‐value theory of achievement emotions (CVTAE), it is postulated that a mindset or belief affects a person's momentary emotions and that there is a reciprocal effect of emotions on beliefs (see Figure 1). The literature on dealing with mistakes shows that, to date, few studies have focused on how people – in this case, students – deal with mistakes and the emotions they experience in the process (e.g., Tulis et al., 2018; Zhao, 2011). In the present study, we focused on three very common emotions experienced in the school context: enjoyment, anxiety and boredom (Zaccoletti et al., 2020). This choice allows for a comparison of the interaction of learning orientation with positive and negative emotions.

The results of a study by Köpfer (2022) suggest that, in practice, teachers' handling of student errors still needs improvement as the learning potential of the error is often not realized. Therefore, we developed an intervention to explore whether students' error learning orientation could be fostered in a school‐like learning situation (Schmid, 2024). The setting of our study was a 1‐day course for visual programming, aimed at secondary school students, which provides an optimal learning context for learning from errors. Our study expands our still modest knowledge of the interplay between error learning orientation and parallel emotional processes in school learning situations.

### Error learning orientation

In school settings, the term error learning orientation refers to whether or not students understand errors as learning opportunities (Spychiger et al., 2006; Tulis et al., 2018). Tulis et al. (2018) use the term ‘beliefs about errors’ for the same construct. This belief has a significant influence on reactions to errors and how errors are learnt from. In terms of content, Spychiger et al. (2006, p. 96) place error learning orientation in the cognitive area as ‘good strategies and intensity of dealing with errors’, ‘willingness to make errors’ or ‘assessment of the significance of errors’. In the school context, a positive error learning orientation can also play a detrimental role in the pursuit of set learning goals and the resulting negative evaluation by teachers, namely poor grades. Accordingly, it is a common practice for students to hide mistakes during the learning process (Soncini et al., 2020). In a recent study, Pan et al. (2020) discovered that even if undergraduate students possessed a positive error learning orientation, they preferred to learn from the correct answers instead of errors. This is another obstacle to utilizing mistakes in the learning process.

Hence, the error learning orientation of students is affected by various variables, such as the teacher's and classmates' behaviour (Spychiger et al., 2006), the learning situation (Meyer et al., 2006) and the learning context and culture (Spychiger et al., 2006). Teachers and classmates play a crucial role in maintaining a positive error learning orientation by responding in an error‐friendly manner, avoiding the mockery of students' errors. According to Hascher and Hagenauer (2010), students describe the undesirable behaviour of classmates as being much more stressful than undesirable teacher behaviour. In relation to the learning situation, a clear distinction between learning and performance situations is essential, as errors in performance situations are perceived less as learning opportunities (Dweck, 2006; Meyer et al., 2006). With regard to the learning context, a distinction should be made among the place of learning, the subjects, the learning materials, the learning method, the learning time and the learning requirements (Brown & Campione, 1996; Chong, 2021; Tulis et al., 2016). In relation to culture, the country where the study on error learning orientation was conducted should be taken into consideration, as different countries may have different error cultures (Spychiger et al., 2006).

To sum up, it can be assumed that a student's error learning orientation has been shaped and fixed to a great extent based on their experiences during their school years. These experiences might have impaired one's own feeling of competence and self‐efficacy in relation to certain topics or subjects (Vongkulluksn et al., 2018). The external attributions of incompetence by teachers or classmates might even have reinforced these negative emotional consequences (Rausch et al., 2017).

### Error learning orientation and emotions

By their very nature, errors are emotional events (Zhao, 2011) because errors are a kind of external feedback about one's own performance. This external performance feedback triggers achievement emotions (Pekrun, 2014), which can be interpreted as internal or internalized performance feedback. Various studies have shown that motivation and beliefs about errors influence self‐regulation strategies in dealing with errors (e.g., Reindl et al., 2020; Tulis et al., 2018). For example, the belief that errors represent learning opportunities (error learning orientation) has an influence on the regulation of negative emotions, experienced as a consequence of an error (Zhang & Fiorella, 2023). Tulis and Ainley (2011) explored the relationship between error learning orientation and state emotions after success and failure. They discovered that positive attitudes towards making mistakes are linked with experiencing interest, enjoyment and pride during successful tasks, and even during tasks in which students have not performed well.

Other studies conclude that emotions form the basis of learning from errors (e.g., Tulis & Dresel, 2018). This is because emotions regulate cognition, motivation and behaviour, which have an important function in constructive learning from errors (Rothermund & Eder, 2009).

Pekrun et al. (2006) established the CVTAE to describe the emotional processes that can occur in learning and achievement situations. The CVTAE also provides a framework for analysing both the influences and effects of emotions. In short, the model explains that the environment influences control and value appraisals, which, in turn, influence emotions and have an impact on learning and performance. In the CVTAE by Pekrun et al. (2006), the error learning orientation can be used as a factor to explain the relationships between the appraisal of the learning situation and the experienced emotions. Therefore, error learning orientation formed part of the achievement goals and beliefs in this study (Figure 1). According to Pekrun et al. (2011), achievement emotions should include positive emotions such as hope, pride and joy, as well as negative emotions like anxiety, shame, boredom, anger and hopelessness. In our study, we chose the three emotions of enjoyment, anxiety and boredom because these three emotions are the most common emotions experienced in a school context, and the three most important emotions that students experience in achievement situations (Goetz & Hall, 2014; OECD, 2017; Raccanello et al., 2018; Zaccoletti et al., 2020).

According to Pekrun et al. (2006) and Pekrun et al. (2018), a positive attitude to errors can reduce negative emotions and promote positive emotions. It can be assumed that students who deal positively with errors and view errors as a learning opportunity will experience negative emotions less strongly or will experience more positive emotions than students who do not view errors as a learning opportunity. However, due to the reciprocal arrows in the CVTAE model, it can be expected that, in addition to the effect of error learning orientation on emotions, emotions can also influence error learning orientation.

The following section describes why our learning environment forms a suitable context for investigating the relationships between error learning orientation and emotions.

### Digital‐based learning environments and dealing with errors

Digital‐based learning environments, such as visual programming with Microsoft MakeCode for micro: bit, offer ideal conditions to promote a positive approach to errors because, in these learning environments, many errors inevitably happen. The micro: bit is a small programmable computer device (4 cm × 5 cm) with a 5×5 LED display, user‐programmable buttons, sensors, Bluetooth technology and expandability. It facilitates technical engagement and programming entry, allowing the easy creation of simple programs without prior coding knowledge (Ball et al., 2016; Schmidt, 2016). The micro: bit is supported by Microsoft MakeCode (https://www.makecode.com) ‘a platform and accompanying web app for simplifying the programming of microcontroller‐based devices in the classroom’ (Ball et al., 2019, p. 7). In this programming environment, the micro:bit can be programmed in the visual or block‐based programming language, Blockly, where existing blocks are put together like pieces of a puzzle, or in the text‐based programming language, JavaScript (Ball et al., 2016; Schmidt, 2016).

While programming the micro:bit, errors often become immediately visible when the micro:bit does not work or does not function as it should. Hence, the students do not get an explicit error message from Microsoft MakeCode. If the micro:bit does not provide an output or functions incorrectly, the students know they have made an error somewhere. The advantage of this is that the students do not have to ask the teacher if the solution is correct, nor do they have to compare their solution with a sample solution to get the answer (Schmid et al., 2022). To support students by learning from errors and to promote a positive error culture, the trial‐and‐error method can be used in the form of debugging tasks. During debugging tasks, faulty tasks are improved; this means that teachers give students tasks to improve (Michaeli & Romeike, 2019; Perscheid et al., 2016). Teachers can use concrete examples of critical thinking in debugging to show students how to better assess their error‐handling skills and build confidence when making mistakes (DeLiema et al., 2020). Debugging tasks can be especially motivating for students who make many errors. This is because, according to Schumacher (2008), working on the errors of others is motivating for students who make many errors. Such tasks often take the form of an experiment. In each experiment, a variable is deliberately changed, and the effect of this change is observed. In this way, learning from trial and error is intended to lead students to the end goal (Bei et al., 2013; Edwards, 2004). By using the trial‐and‐error method, students not only learn how something should be but also how something should not be. The knowledge of how something should not be is called negative knowledge. Knowing if something is wrong or should not be executed is not necessarily what is desired, but this helps prevent students from making the same error again (Oser et al., 1999).

### The present study

This study is part of a larger project, aimed at fostering students' error learning orientation, as well as promoting the understanding of errors in the acquisition of scientific knowledge during a course in visual programming. As part of an intervention, a constructive approach to mistakes was encouraged and students were informed how errors play an important role in scientific experimenting and in making new discoveries (nature of science). In addition, we investigated the different context effects when the students were dealing with errors during the programming tasks, for example, the experience of the students' emotions.

Schmid (2024) showed that the intervention had no effect on the error learning orientation, neither directly after the course nor 2 months later. What remained open, however, and related to the context of Pekrun's (2006) control value theory was the role of the students' state emotions while dealing with errors during our visual programming course. There are few intervention studies aimed at promoting error learning orientation, therefore, our knowledge of how state emotions interact with error learning orientation is still limited. As far as we are aware, no such study has yet been conducted on the subject of computer science.

Therefore, in this study, we examined to what extent momentary emotions, specifically enjoyment, anxiety and boredom, as well as the error learning orientation of students, interacted in a quasi‐experimental setting. In this context, gender and age were taken into account as control variables.

We postulated that:

1. Error learning orientation predicts students' state emotions at the beginning of the course and over time. Specifically, error learning orientation positively predicts the students' state of enjoyment (a) and negatively predicts the students' state of anxiety (b) and boredom (c) at the beginning of the course and over time.
2. State emotions predict students' error learning orientation at the end of the course. Specifically, the state of enjoyment (a) positively predicts the students' error learning orientation, and the states of anxiety (b) and boredom (c) negatively predict the students' error learning orientation.
3. Students' positive emotions in the intervention group increased to a greater extent than those in the control group during the 1‐day course. Specifically, students' enjoyment (a) in the intervention group increased to a greater extent than the enjoyment of those in the control group, and students' anxiety (b) and boredom (c) in the intervention group decreased more than the anxiety and boredom of those in the control group during the 1‐day course.

## METHOD

### Research design

The data for this study were collected from October 2020 to February 2021 in the context of an out‐of‐school learning setting, focusing on *Creativity in Science and Technology – Smart Textiles* at the Swiss education lab, *Smartfeld* (www.smartfeld.ch). The workshop, *Creativity in Science and Technology – Smart Textiles*, was a full‐day course during which lower secondary school students were introduced to programming by solving scientific and technical problems, using the visual programming language, Microsoft MakeCode for micro:bit. The aim of the workshop was to develop different ‘smart textiles’. A smart textile contains a 16×16 LED matrix applied to a T‐shirt and is linked to touch, light and temperature sensors. The students solved tasks such as programming a messenger that communicates by flashing simple LEDs (T1), debugging an LED strip with 10 LEDs (T2) and developing flags for the 16×16 LED matrix, which consisted of 256 LEDs. (T3) is a weather indicator shirt that reacts to light intensity and displays different motifs according to different light intensities and (T4) is a cycling shirt similar to that created by the Ford Media Center (2020), which flashes on the left or right when turning and shows a warning sign when a sensor reacts to the vibrations.

For the study, an intervention was applied. A control group attended the regular workshop, while the students in the intervention group dealt with their errors as follows. At the beginning of the workshop, the intervention group was introduced to three types of errors: material errors, observation errors and programming errors. A four‐step method, adapted from Oser et al. (1999) was then used at each of the four time points (T1–T4), which aimed to promote a constructive approach to errors among the students. With this four‐step method, the students had to find errors (1), analyse them (2) and improve them (4). Between steps 2 and 4, errors as part of scientists' typical approach to working were discussed (3).

Directly before and after the workshop, the students filled out a questionnaire. In addition to information on the students' background, age and gender, this questionnaire also contained items relating to their error learning orientation. Before the four‐step method, the students were also asked four times about their emotions, specifically enjoyment, anxiety and boredom, using the experience sampling method (ESM). ESM is a survey method in which people are randomly asked about their activities, thoughts or feelings during the day. In our study, an acoustic signal was sounded at approximately equal intervals (tasks 1–4) to assess the emotions on a scale. The advantages of the ESM method are that repeated, prospective and ecologically valid measurements can be made (Csikszentmihalyi & Larson, 1987; Fisher & To, 2012; Goetz et al., 2016) as emotions can change from task to task throughout the course day.

### Sample

The sample consisted of 269 lower secondary school students (grades 7–9) from 16 classes. Most of the students were in Grade 8. The average age was 13.6 years and 45% of the students were girls. The students were beginners in programming a visual programming language. The sample was split into two conditions in a quasi‐experimental fashion by assigning complete classes to either the intervention group (129 students) or the control group (140 students). The two groups were divided as equally as possible according to gender, age and school level. All teachers were briefed about the study when they registered for the course. They informed their students that the survey was voluntary and that all data would be anonymized afterwards. The survey was carried out according to the internal guidelines set by the Ethics Committee of the university conducting the research.

### Instruments

#### Questionnaire

##### Traits – error learning orientation

In order to assess the students' error learning orientation, we adopted the error learning orientation scale from Spychiger et al. (2006). The adjustments that have been made can be found in Appendix A1. A 6‐point, balanced, bipolar agreement rating system was used (6 = absolutely agree, 5 = agree, 4 = somewhat agree, 3 = somewhat disagree, 2 = disagree and 1 = absolutely disagree). Item means, SD and reliability values for T1 and T2 are presented in Appendix A2. A confirmatory factor analysis showed good‐fit values at T1: *χ* ^2^ = 17.86, *df* = 5, *p* = .003, RMSEA = .03, CFI = .99, SRMR = .03; and satisfying fit values at T2: *χ* ^2^ = 5.88, *df* = 5, *p* = .32, *χ* ^2^/*df* = 1.17, RMSEA = .10, CFI = .93, SRMR = .05 (Hu & Bentler, 1999).

##### States – emotions

The three emotions of enjoyment, anxiety and boredom were collected as momentary or state emotions with ESM. In accordance with the suggestions of Goetz et al. (2010), all emotions were collected with a single item within ESM. A similar 6‐point, balanced, bipolar agreement rating system for error learning orientation was used. Since the emotions of enjoyment, anxiety and boredom were assessed with single items, no measurement invariance check was carried out.

### Data analysis

A descriptive analysis of the student data was conducted using SPSS. Since error learning orientation can vary from class to class, we additionally estimated the intraclass differences for the participating classes using intraclass coefficients (ICCs). The ICCs of the variables studied were very close to 0 (between .023 and .088), which, according to Hox (2010), meant that there was little variance between the classes and that no multilevel analyses needed to be conducted.

Since the confirmatory factor analysis of the error learning orientation at T2 was less perfect than at T1, a measurement invariance test with MPlus 8.10 was carried out (see Appendix A3).

Structural equation modelling was applied to analyse the development of emotions and beliefs. Specifically, several latent growth curve (LGC) models, according to Bollen and Curran (2006), were computed to compare the development of state emotions, specifically enjoyment, anxiety and boredom over time, with gender, age, intervention group and error learning orientation as covariates. The fit indices of the LGC models were reviewed to test whether the models were appropriate (Hu & Bentler, 1999). Since missing values were not due to the study design, we assumed that they occurred by chance. Consequently, we used the maximum‐likelihood method with complete information (FIML), which means that missing values were taken into account in the model. The number of missing values was less than 10% in all cases, which is acceptable (Enders, 2010).

## RESULTS

In the following, a descriptive overview of the quantitative data used for this study is shown in Table 1. There are small differences between the control group and the intervention group in terms of the mean values. Smaller significant differences were found for anxiety. The students' error learning orientation decreased slightly towards the end of the course in both groups. In relation to the three emotions studied, it can be seen that the state of enjoyment increased, and the state of anxiety and state of boredom declined over the 1‐day workshop across both groups (Table 1).

**TABLE 1:** Overview of scale values at the different points in time.

	CG (*N* = 129)		IG (*N* = 140)		*t*‐test	*d*
	M	SD	M	SD		
Error learning orientation T1	4.33	.73	4.44	.77	−1.19	−.15
Error learning orientation T2	4.18	.93	4.21	.96	−.32	−.03
Enjoyment T1.1	4.20	1.37	4.57	1.24	−2.29	−.28
Enjoyment T1.2	4.31	1.49	4.76	1.30	−2.57*	−.32
Enjoyment T1.3	4.72	1.18	4.81	1.44	−.55	−.07
Enjoyment T1.4	4.84	1.17	5.00	1.23	−1.06	−.43
Anxiety T1.1	2.05	1.20	1.78	.97	1.98**	.25
Anxiety T1.2	1.83	1.07	1.55	.98	2.20*	.27
Anxiety T1.3	1.79	1.20	1.10	.82	2.94***	.68
Anxiety T1.4	1.57	.94	1.29	.72	2.70***	.34
Boredom T1.1	2.69	1.49	2.43	1.52	1.38	.17
Boredom T1.2	2.47	1.50	2.32	1.20	.79	.11
Boredom T1.3	2.17	1.31	2.22	1.69	−.28**	−.03
Boredom T1.4	2.26	1.36	2.02	1.53	1.31	.17

*Note*: Six‐point, balanced, bipolar agreement rating system used for error learning orientation and enjoyment, anxiety and boredom (6 = strongly agree, 5 = mostly agree, 4 = somewhat agree, 3 = somewhat disagree, 2 = mostly disagree and 1 = strongly disagree); T1 = pre‐test, T2 = post‐test, T1.1 = ESM time 1, T1.2 = ESM time 2, T1.3 = ESM time 3, T1.4 = ESM time 4; CG = control group, IG = intervention group; M = mean; SD = standard deviation; effect size according to Cohen (*d*): 0.2 = weak effect, 0.4 = medium effect, 0.8 = strong effect (Cohen, 1988); ***p < .001, **p < .01 and *p < .05.

Table 2 shows the correlation analyses between the scales' error learning orientation, the state of enjoyment, the state of anxiety and the state of boredom in the control and intervention groups at the different time points. There are small differences between the control group and the intervention group in terms of the correlations. The correlations in the control group tended to be slightly higher than in the intervention group. Some correlations were found in one group only. The error learning orientation showed a high and moderately significant correlation between the two time points, T1 and T2, in both groups. Enjoyment, anxiety and boredom also indicated medium‐to‐high and moderately significant correlations between the four time points in both groups. Only in the case of anxiety in the intervention group at time point 1 and time point 3 did the correlation show a low and significant effect.

**TABLE 2:** Correlations of the examined scales at the different time points for the intervention and control groups.

Variable	*n*	1	2	3	4	5	6	7	8	9	10	11	12	13	14
1. Error learning orientation T1	268	–	.497**	.234**	.235**	.194*	.190*	.015	−.028	−.118	−.056	−.122	−.169*	−.090	−.135
2. Error learning orientation T2	240	.686**	–	.170	.340**	.309**	.237*	.107	.021	−.033	−.073	−.267**	−.231*	−.271**	−.213*
3. Enjoyment T1.1	261	.330**	.190*	–	.673**	.613**	.575**	−.176*	−.120	−.159	−.165	−.685**	−.600**	−.488**	−.483**
4. Enjoyment T1.2	257	.297**	.175	.616**	–	.695**	.590**	−.060	−.113	−.151	−.088	−.643**	−.710**	−.625**	−.444**
5. Enjoyment T 1.3	261	.310**	.249**	.581**	.539**	–	.610**	−.026	−.054	−.143	−.076	−.623**	−.573**	−.741**	−.535**
6. Enjoyment T 1.4	259	.425**	.215*	.564**	.632**	.584**	–	−.096	−.067	−.262**	−.101	−.499**	−.427**	−.527**	−.794**
7. Anxiety T 1.1	261	−.112	−.092	−.143	−.174	−.065	−.098	–	.581**	.282**	.451**	.042	.017	−.032	.075
8. Anxiety T 1.2	257	−.031	−.106	−.149	−.178	−.121	−.186*	.625**	–	.572**	.523**	.071	.134	.098	.145
9. Anxiety T 1.3	260	−.162	−.101	−.248**	−.299**	−.308**	−.250**	.442**	.511**	–	.540**	.049	.112	.281**	.283**
10. Anxiety T 1.4	257	−.261**	−.193*	−.227*	−.266**	−.259**	−.337**	.509**	.495**	.579**	–	.045	.022	.036	.161
11. Boredom T 1.1	261	−.324**	−.284**	−.734**	−.503**	−.495**	−.417**	.057	.119	.228*	.144	–	.614**	.532**	.434**
12. Boredom T 1.2	259	−.421**	−.262**	−.537**	−.768**	−.523**	−.575**	.125	.112	.260**	.287**	.629**	–	.629**	.488**
13. Boredom T 1.3	258	−.295**	−.195*	−.526**	−.517**	−.830**	−.590**	.078	.160	.338**	.334**	.558**	.574**	–	.632**
14. Boredom T 1.4	257	−.281**	−.073	−.396**	−.435**	−.433**	−.680**	.114	.170	.084	.206*	.414**	.524**	.496**	–

*Note*: 6‐point, balanced, bipolar agreement rating system used for error learning orientation and enjoyment, anxiety and boredom (6 = strongly agree, 5 = mostly agree, 4 = somewhat agree, 3 = somewhat disagree, 2 = mostly disagree and 1 = strongly disagree); lower diagonal = control group; upper diagonal = intervention group. T1 = pre‐test, T2 = post‐test, T1.1 = ESM time 1, T1.2 = ESM time 2, T1.3 = ESM time 3 and T1.4 = ESM time 4.

Furthermore, significant correlations of varying strength were found between the error learning orientation at T1 and the emotions in both groups. In the intervention group, no significant correlations for error learning orientation were found in relation to anxiety at all four time points or for boredom at T1, T2 or T3. In the control group, no significant correlations for error learning orientation were found in relation to anxiety at T1, T2 and T3. The error learning orientation at T2 also correlated significantly to varying degrees with emotions. In the intervention group, the error learning orientation at T2 did not correlate with enjoyment at T1 and anxiety at all four time points. In the control group, error learning orientation at T2 did not correlate with enjoyment at T2 and anxiety at T1, T2 and T3.

In order to clarify the extent to which students' momentary emotions, namely enjoyment, anxiety and boredom interacted with error learning orientation in a quasi‐experimental setting; different LGC models were constructed. First, the linear and quadratic LGC models were compared separately for enjoyment, anxiety and boredom. For all three emotions, the linear model offered better‐fit values. In the next step, the covariates of gender, age and intervention were added. As a last step, the pre‐ and post‐error learning orientation was included. Thus, error learning orientation was a predictor and an outcome in each of the three models. The final models also showed satisfactory fit values (Table 3).

**TABLE 3:** Fit values for the LGC for enjoyment, anxiety and boredom.

	*χ* ^2^	*df*	*p*	*χ* ^2^/*df*	RMSEA	CFI	SRMR
LGC for enjoyment	92.26	51	<.001	1.80	.06	.96	.06
LGC for anxiety	76.98	51	<.01	1.51	.04	.97	.05
LGC for boredom	99.00	51	<.001	1.94	.06	.94	.06

Abbreviations: CFI, comparative fit index; *df*, degrees of freedom; LGC, latent growth curve model; RMSEA, root mean square error of approximation; SRMR, standardized root mean square residual; *χ* ^2^, Chi‐square.

**FIGURE 2:**
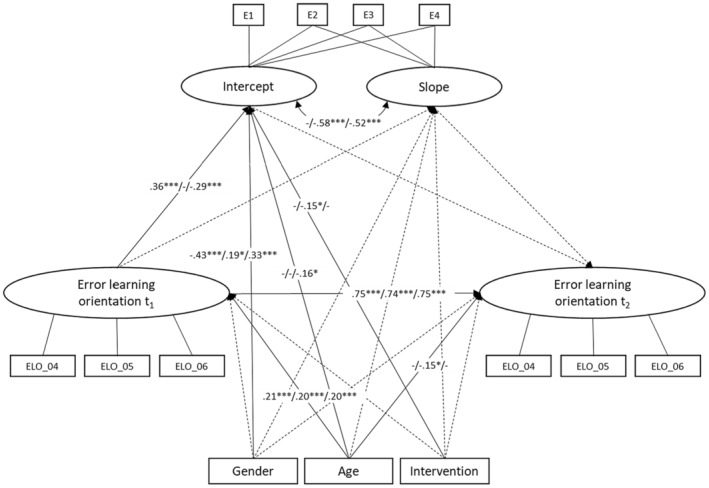
LGC model for enjoyment/anxiety/boredom over time, with gender, age, intervention group and error learning orientation as covariates. Standardized estimates; *p < .05, **p < .01, ***p < .001, intercept = initial value of emotions, slope = change of emotions over the course, E1–E4 = emotions (enjoyment/anxiety/boredom) at T1–T4.

The results of the LGC (Figure 2, Appendix A4) indicated that the students' error learning orientation at time, T1, had a significant effect on the initial value (intercept) of enjoyment (β = .36, *p* < .001) and boredom (β = −.29, *p* < .001) but not on anxiety (β = −.08, *p* = .28). Students with a more positive error learning orientation reported more enjoyment and less boredom. However, the error learning orientation at T1 had no significant effect on how enjoyment (β = .01, *p* = .95), anxiety (β = −.11, *p* = .34) or boredom (β = .02, *p* = .87) developed over the course of the workshop (slope). Therefore, Hypotheses 1a and 1c were partially confirmed and Hypothesis 1b was rejected.

Neither the initial value of enjoyment (β = .03, *p* = .90), anxiety (β = −.05, *p* = .71) and boredom (β = −.04, *p* = .79), nor the change in enjoyment (β = .03, *p* = .94), anxiety (β = −.11, *p* = .51) and boredom (β = .24, *p* = .18) over time had an influence on the students' error learning orientation at the end of the course. This indicates that the states of enjoyment, anxiety and boredom did not predict students' error learning orientation at the end of the course. Thus, Hypotheses 2a, 2b and 2c were rejected.

The intervention group had no effect on the change in enjoyment (β = −.18, *p* = .42), anxiety (β = .01, *p* = .95) and boredom (β = .02, *p* = .84) during the workshop. Therefore, Hypotheses 3a, 3b and 3c were rejected.

However, the results showed that the anxiety intercept (β = −.58, *p* < .001) and the boredom intercept (β = −.52, *p* < .001) correlated negatively with the slope. This means that the anxiety or boredom of students, who experienced heightened anxiety or more boredom at the start of the course, decreased less than that of students who had less anxiety or less boredom.

In addition, the results showed that age had, in all three cases, a small significant effect on error learning orientation at T1 (enjoyment: β = .21/anxiety: β = .20/boredom: β = .21, *p* < .001; see Figure 1), indicating that older students had a more positive error learning orientation at the beginning of the course than younger students. In the case of anxiety, age also had a small negative significant effect on error learning orientation at T2 (β = −.15, *p* < .01), indicating that older students had a less positive error learning orientation than younger students at the end of the course. In the case of boredom, age also had a negative significant effect on the initial value of boredom (intercept) (β = −.16, *p* < .05). This means that older students were less bored than younger students at the beginning of the course.

Gender also had an effect on the initial value of enjoyment (β = −.43, *p* < .001), anxiety (β = .19, *p* < .01) and boredom (β = .33, *p* < .001). In our case, females exhibited less enjoyment and more anxiety and were more bored at the beginning of the course than males.

Furthermore, the error learning orientation at T2 was, in all three models, strongly dependent on the error learning orientation at T1 (β = .75/β = .74/β = .75, *p* < .001; see Figure 2).

The covariate intervention had a significant effect only on the initial value (intercept) of anxiety (β = −.15, *p* < .05) but not on enjoyment and boredom. Therefore, students in the control group experienced more anxiety than students in the intervention group.

The three models explained between 55.9% and 62.6% of student variance in terms of their error learning orientation at the end of the workshop, with a total beta effect of .76 (*p* < .001) for each. The direct effect of error learning orientation at T1 on error learning orientation at T2 was strong and significant (β = .75/β = .74/β = .75, *p* < .001, 95% CI [.56/.62/.61–.99/.89/.92]). The indirect effects of error learning orientation at T1 on error learning orientation at T2 via emotions were tested, but no significant results were found.

## DISCUSSION

The research question examined to what extent the state emotions of enjoyment, anxiety and boredom interacted with students' error learning orientation over the course of a day in a quasi‐experimental setting. Overall, it appeared that error learning orientation was relatively stable, while positive emotions became stronger and negative emotions diminished.

According to the CVTAE of Pekrun et al. (2006), it was proposed that the error learning orientation predicted students' state emotions at the start and over time (Hypothesis 1). In our study, Hypothesis 1 could be partly confirmed. The error learning orientation at T1 had an effect on students' enjoyment and boredom at the beginning of the workshop. This means that students with a positive error learning orientation, that is, students who view errors as learning opportunities, experienced more enjoyment and less boredom with the first programming task. This result is in line with the findings of Zhang and Fiorella (2023), who state that the belief that errors represent learning opportunities has an influence on the regulation of negative emotions experienced as a consequence of an error. It also confirms the assumption of Pekrun et al. (2006) and Pekrun et al. (2018) that a positive attitude to errors not only reduces negative emotions but also promotes positive emotions. However, no effect of error learning orientation on students' anxiety at T1 in relation to the first programming task or on the change in the three emotions across the four tasks was found. It seems that in the case of anxiety, some students are generally anxious in performance situations independently of the events occurring in a particular situation. The nature of the anxiety trait is related to attitudes, and it is not linked to situational triggers (Pacheco‐Unguetti et al., 2010). It showed that, in the case of highly anxious students, values dropped less than for less anxious students. Overall, however, it could be assumed that our extracurricular learning place and learning environment for visual programming reduced error anxiety. What remains unclear is why no relationship was found between the learning orientation at T1 and the development of the emotions over time (slope). It seems that the relationship between emotions and error learning orientation became weaker over the four tasks. It is difficult to compare our results with results from other studies (e.g., Tulis & Ainley, 2011; Tulis & Dresel, 2018; Zhang & Fiorella, 2023) because those emotions were measured as traits and not as states in an experience sampling setting. Since the intervention also had no significant effect on the development of emotions, it can be assumed that another factor, such as the development of self‐efficacy beliefs, could have a greater influence on the development of emotions than error learning orientation (e.g., Putwain et al., 2013). According to the CVTAE from Pekrun et al. (2006), many other factors can affect emotions or emotional development, which were not examined in this study.

Similar to Hypothesis 1, it was expected that state emotions predicted students' error learning orientation at the end of the course (Hypothesis 2). In the case of Hypothesis 2, the results were different. Students with more positive emotions at the beginning of the course did not have a more positive error learning orientation at the end of the course. In addition, those displaying an increased growth in positive emotions or a greater decrease in negative emotions tended not to possess a more positive learning orientation at the end of the course. The results in our study seem to contrast with the statement of Rothermund and Eder (2009), which implies that emotions regulate cognition, motivation and behaviour, all three having an important function in terms of constructive learning from errors.

Furthermore, the reciprocal effect according to the CVTAE of Pekrun et al. (2006) could not be confirmed: emotions were partly influenced by the students' antecedent error learning orientation but, in turn, had no influence on the postcedent error learning orientation. Hence, Hypothesis 2 was rejected. Nevertheless, the emotions could have had an influence on other aspects related to dealing with errors, for example, action adaptability (Dresel et al., 2013) or factors such as motivation to learn (Pekrun, 2006), which were not surveyed in this study. However, considering that error learning orientation has rather high stability, it is positive that the students' emotions at the end of the day no longer exhibited any relationship with error learning orientation. This means that the students who experienced considerable boredom and minimal enjoyment no longer had a significantly low error learning orientation at the end of the day.

In addition, it was assumed that the positive emotions of students in the intervention group would increase more, and the negative emotions would decrease more than those of students in the control group (Hypothesis 3). Hypothesis 3 was mostly rejected as only an effect of the intervention on the initial value of anxiety was found. It could be assumed that the discussion of the error types at the beginning of the workshop with the intervention groups eased some of the anxiety of the students in this group. No effect of the intervention on the initial values of enjoyment and boredom or the increase in the three emotions could be demonstrated. It must be assumed that the intervention was not suitable or too short for these emotions.

What influences did the age and gender covariates have? Age had an effect on error learning orientation at T1, meaning that older students possessed a more positive learning orientation at the beginning of the course but not at the end. This suggests that the greater schooling experience of older students made a positive contribution to error learning orientation. It may also be the case that the older students had greater academic success and had learned from prior assessment opportunities. Gender had an effect on emotions. Boys showed a greater number of positive emotions and fewer negative emotions at the beginning of the course than girls. The tendency that boys experienced more positive and fewer negative emotions regarding errors in this study is consistent with the findings of Dresel et al. (2013) and Schmid et al. (2022). According to Tellhed et al. (2023), it could also be assumed that girls believe that boys are better at computer science and therefore devalue themselves at the beginning and over the course. Although not significant, the values indicate that our learning environment might have positively supported boys' as well as girls' emotional experiences, which are ultimately connected to learning and motivation. Both are important aspects from the perspective of wanting to deal with errors.

### Strengths, limitations and future research

This study used LGC with data from secondary school students from Switzerland showing to what extent momentary emotions, specifically enjoyment, anxiety and boredom, as well as the error learning orientation of students, interacted over a 1‐day course on visual programming in an out‐of‐school learning environment. We found significant effects of error learning orientation on students' emotions at the beginning of the course. These clear relationships became vaguer as the course progressed resulting in no further significance at the end. This was accompanied by the result that the emotions changed positively over the course of the workshop, while the error learning orientation remained stable. The fact that we considered the current emotions in a longitudinal setting, not just at one point, and conducted an intervention is a strength of this study. In addition, we were able to provide a further corroborating result that the error learning orientation is not easy to change and that different emotions present specific interactions.

Nonetheless, this study has several limitations in various areas that may have influenced the results. It must be noted that our study involved beginners in visual programming. Different results might have been obtained had advanced learners participated. The latter might be used to the rather large number of errors in visual programming and may view them more as a learning opportunity than beginners. Another limitation is the rather small sample size, which resulted from the fact that the data were collected during the COVID‐19 pandemic and many workshops were cancelled. These results should be compared with other studies with larger samples to determine whether similar or different results are recorded when dealing with errors in visual programming environments. Further limitations are that measurement errors cannot be modelled with single items, such as those used for the three emotions. In addition, no reliability tests can be carried out with single items. Another limitation is that the autocorrelations of emotions were not explicitly modelled.

We chose the three emotions, namely enjoyment, anxiety and boredom, as these occur most frequently when solving tasks according to Zaccoletti et al. (2020). Further investigation is required to determine whether the same or different results are obtained with other emotions, for example, frustration (Coto et al., 2022). A comparison with other learning places, subjects or topics would also be necessary. In any case, we recommend choosing tasks that invite students to make errors.

There are also limitations with regard to the scale error learning orientation. We were only able to use three of seven items on the scale. According to Spychiger, Kuster und Oser (2006, p. 96), error learning orientation in the cognitive area contains ‘good strategies and intensity of dealing with errors’, ‘willingness to make errors’ or ‘assessment of the significance of errors’. The items 4–6 used related to positive attitude to learn from errors (L‐4 & L‐5) and willingness to learn from errors (L‐6). The two items L‐2 and L‐7 had to be excluded after a measurement invariance test. L‐2 could be worded unfavourably, for example, because of the introduction with sometimes. L‐7 could be different/not appropriate because it addresses emotions and not just cognition. The three remaining items partially confirm the factors of Spychiger et al. (2006). The question arises as to whether something has changed in the attitudes of pupils in the 20 years or so since the scales were constructed and whether a revision of the scales is necessary.

We assume that students with a positive error learning orientation experience enjoyment and no boredom and anxiety even if they make a lot of errors. To clarify this, actually we should have asked the students at the end whether they made many or few errors. This would also be a suggestion for further research.

## CONCLUSION

Several factors can influence students' error learning orientation, the effects of which have not yet been researched extensively. Emotions were shown to have a significant relationship with error learning orientation, but error learning orientation appeared to be a rather stable concept that could not easily be changed and needed more time. These findings raise the question of whether teachers should pay more attention to the development of students' error learning orientation by addressing the topic more explicitly in daily lessons. In addition, it might be beneficial to provide students with knowledge of how errors can contribute positively to learning (Pan et al., 2020).

There was a general decline in negative emotions in our learning setting. It was assumed that the reduction of negative emotions when making errors, in combination with a positive error culture over a longer period of time, could have had an influence on the error learning orientation (Spychiger et al., 2006). It is probable that students must undertake more learning experiences before emotions have a positive effect on their error learning orientation. This means teachers have to be patient because the error learning orientation does not change particularly quickly.

Through repeated, targeted, pedagogical approaches that aim to minimize the number of negative emotions that occur while promoting an understanding of the constructive role of errors, teachers could help create a positive learning environment in which errors are seen as a natural part of the learning process. This applies, in particular, to more problem‐orientated learning activities in the field of computer science. It would be interesting and recommended in future research to study the development of error learning orientation in connection with emotions over a longer period, for example, one or more school years.

## AUTHOR CONTRIBUTIONS

**Rahel Schmid:** Conceptualization; investigation; writing – original draft; methodology; validation; visualization; writing – review and editing; software; formal analysis; project administration; data curation. **Robbert Smit:** Writing – original draft; conceptualization; methodology; validation; visualization; writing – review and editing; software; formal analysis. **Nicolas Robin:** Funding acquisition; conceptualization; writing – original draft; writing – review and editing; supervision. **Alexander Strahl:** Supervision; writing – original draft.

## CONFLICT OF INTEREST STATEMENT

None to declare.

## Supporting information


Data S1

## Data Availability

The data that support the findings of this study are available from the corresponding author upon reasonable request.

## References

[bjep12681-bib-0001] Ball, T. , Chatra, A. , de Halleux, P. , Hodges, S. , Moskal, M. , & Russell, J. (2019). Microsoft MakeCode: Embedded programming for education, in blocks and TypeScript . Paper presented at the proceedings of the 2019 ACM SIGPLAN symposium on SPLASH‐E, Athens, Greece. 10.1145/3358711.3361630

[bjep12681-bib-0002] Ball, T. , Protzenko, J. , Bishop, J. , Moskal, M. , de Halleux, J. , Braun, M. , Hodges, S. , & Riley, C. (2016). Microsoft touch develop and the BBC micro:Bit . Paper presented at the proceedings of the 38th international conference on software engineering companion, Austin, Texas. 10.1145/2889160.2889179

[bjep12681-bib-0003] Bei, X. , Chen, N. , & Zang, S. (2013). On the complexity of trial and error . Paper presented at the proceedings of the forty‐fifth annual ACM symposium on the theory of computing. 10.1145/2488608.2488613?casa_token=XEqMB1DOuvYAAAAA:MxOyo9KPJFwuBOfFK9v5A6DiYjMi5INVALZLanHPvS2UItXaONV3_8An6c5Zl685M47l33Uu8HuTAQE

[bjep12681-bib-0005] Bligha, M. C. , Kohles, J. C. , & Yan, Q. (2018). Leading and learning to change: The role of leadership style and mindset in error learning and organizational change. Journal of Change Management, 18(2), 116–141. 10.1080/14697017.2018.1446693

[bjep12681-bib-0006] Bollen, K. A. , & Curran, P. J. (2006). Latent curve models: A structural equation perspective. Wiley‐Interscience.

[bjep12681-bib-0007] Brown, A. L. , & Campione, J. C. (1996). Psychological theory and the design of innovative learning environments: On procedures, principles, and systems. In L. Schauble & R. Glaser (Eds.), Innovations in learning. Routledge.

[bjep12681-bib-0008] Chong, S. W. (2021). Reconsidering student feedback literacy from an ecological perspective. Assessment & Evaluation in Higher Education, 46(1), 92–104. 10.1080/02602938.2020.1730765

[bjep12681-bib-0009] Cohen, J. (1988). Statistical power analysis for the behavioral sciences (2nd ed.). Routledge.

[bjep12681-bib-0010] Coto, M. , Mora, S. , Grass, B. , & Murillo‐Morera, J. (2022). Emotions and programming learning: Systematic mapping. Computer Science Education, 32(1), 30–65. 10.1080/08993408.2021.1920816

[bjep12681-bib-0011] Csikszentmihalyi, M. , & Larson, R. (1987). Validity and reliability of the experience‐sampling method. The Journal of Nervous and Mental Disease, 175(9), 526–536. 3655778 10.1097/00005053-198709000-00004

[bjep12681-bib-0012] DeLiema, D. , Dahn, M. , Flood, V. J. , Asuncion, A. , Abrahamson, D. , Enyedy, N. , & Steen, F. (2020). Debugging as a context for fostering reflection on critical thinking and emotion. In E. Manalo (Ed.), Deeper learning, dialogic learning, and critical thinking. Innovative research‐based strategies for development in 21st century classrooms (pp. 209–228). Routledge.

[bjep12681-bib-0013] Diener, C. I. , & Dweck, C. S. (1980). An analysis of learned helplessness: II. The processing of success. Journal of Personality and Social Psychology, 39(5), 940–952. 10.1037/0022-3514.39.5.940 7441483

[bjep12681-bib-0014] Dresel, M. , Schober, B. , Ziegler, A. , Grassinger, R. , & Steuer, G. (2013). Affektiv‐motivational adaptive und handlungsadaptive Reaktionen auf Fehler im Lernprozess [Affective‐motivational adaptive and action‐adaptive reactions to errors in the learning process]. Zeitschrift für Pädagogische Psychologie, 27(4), 255–271. 10.1024/1010-0652/a000111

[bjep12681-bib-0015] Dweck, C. S. (2006). Mindset: The new psychology of success. Random House.

[bjep12681-bib-0016] Edwards, S. H. (2004). Using software testing to move students from trial‐and‐error to reflection‐in‐action . Paper presented at the proceedings of the 35th SIGCSE technical symposium on computer science education. 10.1145/971300.971312?casa_token=B6TCinO8-0AAAAAA:NqMbdWSkmyoQ-0ynGKvpsraGBZmJeNC6dR0_C9FepUVh24XvLwnJZd3zOlCoLeixqqHMjPHLPlTcxmg

[bjep12681-bib-0017] Enders, C. K. (2010). Applied missing data analysis. Guilford.

[bjep12681-bib-0018] Fisher, C. D. , & To, M. L. (2012). Using experience sampling methodology in organizational behavior. Journal of Organizational Behavior, 33(7), 865–877. 10.1002/job.1803

[bjep12681-bib-0019] Ford Media Center . (2020). Emoji jacket helps people to ‘Share the road’ . https://media.ford.com/content/fordmedia/feu/en/news/2020/02/06/emoji‐jacket‐helps‐people‐to‐share‐the‐road.html

[bjep12681-bib-0020] Goetz, T. , Bieg, M. , & Hall, N. C. (2016). Assessing academic emotions via the experience sampling method. In M. Zembylas & P. A. Schutz (Eds.), Methodological advances in research on emotion and education. Springer.

[bjep12681-bib-0021] Goetz, T. , Frenzel, A. C. , Stoeger, H. , & Hall, N. C. (2010). Antecedents of everyday positive emotions: An experience sampling analysis. Motivation and Emotion, 34, 49–62. 10.1007/s11031-009-9152-2

[bjep12681-bib-0022] Goetz, T. , & Hall, N. C. (2014). Academic boredom. In R. Pekrun & L. Linnenbrink‐Garcia (Eds.), International handbook of emotions and education (pp. 311–330). Routledge.

[bjep12681-bib-0023] Hascher, T. , & Hagenauer, G. (2010). Lernen aus Fehlern [Learning from errors]. In C. Spiel , B. Schober , P. Wagner , & R. Reimann (Eds.), Bildungspsychologie (pp. 377–381). Hogrefe.

[bjep12681-bib-0025] Hox, J. J. (2010). Multilevel analysis: Techniques and applications (2nd ed.). Routledge.

[bjep12681-bib-0026] Hu, L. , & Bentler, P. M. (1999). Cutoff criteria for fit indexes in covariance structure analysis: Conventional criteria versus new alternatives. Structural Equation Modeling: A Multidisciplinary Journal, 6(1), 1–55. 10.1080/10705519909540118

[bjep12681-bib-0029] Köpfer, P. (2022). Teachers' perspectives on dealing with students' errors. Frontiers in Education, 7, 1–17. 10.3389/feduc.2022.868729

[bjep12681-bib-0030] Kreutzmann, M. , Zander, L. , & Hannover, B. (2014). Versuch macht kluchg?! Der Umgang mit Fehlern auf Klassen‐ und Individualebene. Zusammenhänge mit Selbstwirksamkeit, Anstrengungsbereitschaft und Lernfreude von Schülerinnen und Schüler [Trial makes perfect?! Dealing with mistakes at class and individual level. Connections with self‐efficacy, willingness to make an effort and students' enjoyment of learning]. Zeitschrift für Entwicklungspsychologie Und Pädagogische Psychologie, 46(2), 101–113. 10.1026/0049-8637/a000103

[bjep12681-bib-0031] Mera, Y. , Rodríguez, G. , & Marin‐Garcia, E. (2022). Unraveling the benefits of experiencing errors during learning: Definition, modulating factors, and explanatory theories. Psychonomic Bulletin & Review, 29(3), 753–765. 10.3758/s13423-021-02022-8 34820785

[bjep12681-bib-0032] Metcalfe, J. (2017). Learning from errors. Annual Review of Psychology, 68, 465–489. 10.1146/annurev-psych-010416-044022 27648988

[bjep12681-bib-0033] Meyer, L. , Seidel, T. , & Prenzel, M. (2006). Wenn Lernsituationen zu Leistungssituationen werden: Untersuchung zur Fehlerkultur in einer Videostudie [When learning situations become performance situations: Investigating error culture in a video study]. Schweizerische Zeitschrift für Bildungswissenschaften, 28(1), 21–41.

[bjep12681-bib-0034] Michaeli, T. , & Romeike, R. (2019). Debuggen im Unterricht – Ein systematisches Vorgehen macht den Unterschied [Debugging in the classroom – a systematic approach makes all the difference]. In A. Pasternak (Ed.), Informatik für alle (pp. 129–138). Gesellschaft für Informatik.

[bjep12681-bib-0035] OECD . (2017). Programme for International Student Assessment (PISA). Results from PISA 2015: Students' well‐being . www.oecd.org.edu/pisa

[bjep12681-bib-0036] Oser, F. , Hascher, T. , & Spychiger, M. (1999). Lernen aus Fehlern. Zur Psychologie des "negativen" Wissens [Learning from mistakes. The psychology of “negative” knowledge]. In W. Althof (Ed.), Fehlerwelten. Vom Fehlermachen und Lernen aus Fehlern (pp. 11–41). Springer Fachmedien.

[bjep12681-bib-0037] Pacheco‐Unguetti, A. P. , Acosta, A. , Callejas, A. , & Lupiáñez, J. (2010). Attention and anxiety: Different attentional functioning under state and trait anxiety. Psychological Science, 21(2), 298–304. 10.1177/0956797609359624 20424060

[bjep12681-bib-0038] Pan, S. C. , Sana, F. , Samani, J. , Cookee, J. , & Kim, J. A. (2020). Learning from errors: Students' and instructors' practices, attitudes, and beliefs. Memory, 28(9), 1105–1122. 10.1080/09658211.2020.1815790 32928077

[bjep12681-bib-0039] Pekrun, R. (2006). The control‐value theory of achievement emotions: Assumptions, corollaries, and implications for educational research and practice. Educational Psychology Review, 18(4), 315–341. 10.1007/s10648-006-9029-9

[bjep12681-bib-0040] Pekrun, R. (2014). Emotions and learning. International Academy of Education.

[bjep12681-bib-0041] Pekrun, R. , Elliot, A. J. , & Maier, M. A. (2006). Achievement goals and discrete achievement emotions: A theoretical model and prospective test. Journal of Educational Psychology, 98(3), 583–597. 10.1037/0022-0663.98.3.583

[bjep12681-bib-0042] Pekrun, R. , Goetz, T. , Frenzel, A. C. , Barchfeld, P. , & Perry, R. P. (2011). Measuring emotions in students' learning and performance: The achievement emotions questionnaire (AEQ). Contemporary Educational Psychology, 36(1), 36–48. 10.1016/j.cedpsych.2010.10.002

[bjep12681-bib-0043] Pekrun, R. , Muis, K. R. , Frenzel, A. C. , & Goetz, T. (2018). Emotions at school. Routledge.

[bjep12681-bib-0044] Perscheid, M. , Siegmund, B. , Taeumel, M. , & Hirschfeld, R. (2016). Studying the advancement in debugging practice of professional software developers. Software Quality Journal, 25, 83–110. 10.1007/s11219-015-9294-2

[bjep12681-bib-0045] Putwain, D. , Sander, P. , & Larkin, D. (2013). Academic self‐efficacy in study‐related skills and behaviours: Relations with learning‐related emotions and academic success. British Journal of Educational Psychology, 83(4), 633–650. 10.1111/j.2044-8279.2012.02084.x 24175686

[bjep12681-bib-0046] Raccanello, D. , Hall, R. , & Burro, R. (2018). Salience of primary and secondary school students' achievement emotions and perceived antecedents: Interviews on literacy and mathematics domains. Learning and Individual Differences, 65, 65–79. 10.1016/j.lindif.2018.05.015

[bjep12681-bib-0047] Rausch, A. , Seifried, J. , & Harteis, C. (2017). Emotions, coping, and learning in error situations in the workplace. Journal of Workplace Learning, 29, 374–393. 10.1108/JWL-01-2017-0004

[bjep12681-bib-0048] Reindl, M. , Tulis, M. , & Dresel, M. (2020). Profiles of emotional and motivational self‐regulation following errors: Associations with learning. Learning and Individual Differences, 77(102027), 101806. 10.1016/j.lindif.2019.101806

[bjep12681-bib-0049] Rothermund, K. , & Eder, A. B. (2009). Emotion und Handeln [Emotion and action]. In V. Brandstätter & H. H. Otto (Eds.), Handbuch der Psychologie: Motivation und emotion (pp. 675–685). Hogrefe.

[bjep12681-bib-0050] Schmid, R. (2024). Verständnis von Nature of Science‐Aspekten und Umgang mit Fehlern von Schüler*innen der Sekundarstufe I – Am Beispiel von digital‐basierten Lernprozessen im informellen Lernsetting Smartfeld [Understanding nature of science aspects and dealing with errors of lower secondary school students – using the example of digital‐based learning processes in the informal learning setting Smartfeld]. Logos (Studien zum Physik– und Chemielernen). 10.30819/5723

[bjep12681-bib-0051] Schmid, R. , Robin, N. , Smit, R. , & Strahl, A. (2022). The influence of error learning orientation on intrinsic motivation for visual programming in STEM education. European Journal of STEM Education, 7(1), 5. 10.20897/ejsteme/12477

[bjep12681-bib-0052] Schmidt, A. (2016). Increasing computer literacy with the BBC micro:Bit. IEEE Pervasive Computing, 15(2), 5–7. 10.1109/MPRV.2016.23

[bjep12681-bib-0053] Schumacher, R. (2008). Der produktive Umgang mit Fehlern. Fehler als Lerngelegenheit und Orientierungshilfe [Dealing productively with errors. Errors as a learning opportunity and orientation aid]. In R. Caspary (Ed.), Nur wer Fehler macht, kommt weiter. Wege zu einer neuen Fehlerkultur (pp. 49–72). Herder.

[bjep12681-bib-0054] Soncini, A. , Matteucci, M. C. , & Butera, F. (2020). Error handling in the classroom: An experimental study of teachers' strategies to foster positive error climate. European Journal of Psychology of Education, 36, 719–738. 10.1007/s10212-020-00494-1

[bjep12681-bib-0055] Spychiger, M. , Kuster, R. , & Oser, F. (2006). Dimensionen von Fehlerkultur in der Schule und deren Messung. Der Schülerfragebogen zur Fehlerkultur im Unterricht für Mittel‐ und Oberstufe [Dimensions of error culture at school and their measurement. The student questionnaire on error culture in lessons for middle and upper secondary schools]. Schweizerische Zeitschrift für Bildungswissenschaft, 28, 87–110. 10.25656/01:4140

[bjep12681-bib-0056] Tellhed, U. , Björklund, F. , & Strand, K. K. (2023). Tech‐savvy men and caring women: Middle school students' gender stereotypes predict interest in tech‐education. Sex Roles, 88, 307–325. 10.1007/s11199-023-01353-1

[bjep12681-bib-0057] Tulis, M. , & Ainley, M. (2011). Interest, enjoyment and pride after failure experiences? Predictors of students' state emotions after success and failure during learning in mathematics. Educational Psychology, 31(7), 779–807. 10.1080/01443410.2011.608524

[bjep12681-bib-0058] Tulis, M. , & Dresel, M. (2018). Emotionales Erleben und dessen Bedeutung für das Lernen aus Fehlern [Emotional experience and its significance for learning from errors]. In G. Hagenauer & T. Hascher (Eds.), Emotionen und Emotionsregulation in Schule und Hochschule. Waxmann.

[bjep12681-bib-0059] Tulis, M. , Steuer, G. , & Dresel, M. (2016). Learning from errors: A model of individual processes. Frontline Learning Research, 4(2), 12–26. 10.14786/flr.v4i2.168

[bjep12681-bib-0060] Tulis, M. , Steuer, G. , & Dresel, M. (2018). Positive beliefs about errors as an important element of adaptive individual dealing with errors during academic learning. Educational Psychology, 38(1), 139–158. 10.1080/01443410.2017.1384536

[bjep12681-bib-0061] Vongkulluksn, V. W. , Matewos, A. M. , Sinatra, G. M. , & Marsh, J. A. (2018). Motivational factors in makerspaces: A mixed methods study of elementary school students' situational interest, self‐efficacy, and achievement emotions. International Journal of STEM Education, 5, 43. 10.1186/s40594-018-0129-0 30631733 PMC6310445

[bjep12681-bib-0062] Zaccoletti, S. , Altoè, G. , & Mason, L. (2020). Enjoyment, anxiety and boredom, and their control‐value antecedents as predictors of reading comprehension. Learning and Individual Differences, 79, 1–11. 10.1016/j.lindif.2020.101869

[bjep12681-bib-0063] Zhang, Q. , & Fiorella, L. (2023). An integrated model of learning from errors. Educational Psychologist, 58(1), 18–34. 10.1080/00461520.2022.2149525

[bjep12681-bib-0064] Zhao, B. (2011). Learning from errors: The role of context, emotion, and personality. Journal of Organizational Behavior, 32(3), 435–463. 10.1002/job.696

