# Supplementary material for: The role of momentary emotions in promoting error learning orientation among lower secondary school students: An intervention study embedded in a short visual programming course
Source: Br J Educ Psychol. 2024 Mar 19;95(1):107–23. doi: 10.1111/bjep.12681 (PMC11802965; doi:10.1111/bjep.12681)
Supplement: Supplementary file 1 — Data S1 [file BJEP-95-107-s001.docx]

# Appendix

**A1 Questionnaire**

Traits – error learning orientation

The error learning orientation scale was adapted from Spychiger et al. (2006). This scale contains eight items and is intended for use in the classroom. For our purpose, in the out-of-school learning environment, *Smartfeld*, two items, namely, “L-1: I always voluntarily improve mistakes in exams, even if the teacher does not tell me to do so” and “L-8: I take a close look at mistakes I made during the lesson at home” were excluded, because no exam was taken in the workshop and the post-test took place before the students went home. The item “L-3: I enjoy trying different ways of solving a task” was excluded after the first piloting due to a discriminatory power < .40. Thus, the following five items were adapted from Spychiger et al. (2006, p. 97) for the study:

L-2: Sometimes in class, it helps me to remember a mistake so that I do not make it again.

L-4: If I do something clumsy in class, I take it as an opportunity from which to learn.

L-5: Mistakes in class help me to do better afterwards.

L-6: I reconsider incorrect solutions in tasks several times.

L-7: I enjoy acquiring new knowledge through mistakes.

**A2 Scales**

Items’ scale analyses, “Error learning orientation” T1

| Item |  | *M* | *SD* | *r_itc_* |
| --- | --- | --- | --- | --- |
| L-2(t1) | Sometimes in class, it is helpful to remember an error, so I do not make it again. | 4.43 | 1.03 | 0.44 |
| L-4(t1) | If I do something wrong in class, I use it as an opportunity from which to learn. | 4.46 | 0.99 | 0.58 |
| L-5(t1) | Errors made in class help me improve afterwards. | 4.58 | 0.92 | 0.56 |
| L-6(t1) | I reconsider incorrect solutions in assignments several times. | 4.07 | 1.07 | 0.39 |
| L-7(t1) | I enjoy acquiring new knowledge through errors. | 3.91 | 0.99 | 0.55 |

**Note:** *N* = 250. Six-point, balanced, bipolar agreement rating system; 6 = absolutely agree, 5 = agree, 4 = somewhat agree, 3 = somewhat disagree, 2 = disagree and 1 = absolutely disagree, Cronbach’s α = 0.74

Items’ scale analyses, “Error learning orientation” T2

| Item |  | *M* | *SD* | *r_itc_* |
| --- | --- | --- | --- | --- |
| L-2(t2) | Sometimes in class, it is helpful to remember an error, so I do not make it again. | 4.53 | 1.12 | 0.54 |
| L-4(t2) | If I do something wrong in class, I use it as an opportunity from which to learn. | 4.29 | 1.18 | 0.66 |
| L-5(t2) | Errors made in class help me improve afterwards. | 4.30 | 1.19 | 0.57 |
| L-6(t2) | I reconsider incorrect solutions in assignments several times. | 4.03 | 1.23 | 0.43 |
| L-7(t2) | I enjoy acquiring new knowledge through errors. | 4.30 | 1.17 | 0.54 |

**Note:** *N* = 217. Six-point, balanced, bipolar agreement rating system; 6 = absolutely agree, 5 = agree, 4 = somewhat agree, 3 = somewhat disagree, 2 = disagree and 1 = absolutely disagree, Cronbach’s α = 0.77

**A3 Measurement invariance test for error learning orientation**

A measurement invariance test for error learning orientation was carried out with MPlus 8.10 (Table 1). This was done to check whether the scale error learning orientation measured the same construct at the two time points (Bialosiewicz, Murphy & Berry, 2013; Kleinke, Schlüter & Christ, 2017). As a result of the measurement invariance test, two further items, namely items L-2 and L-7, had to be excluded as not even metric invariance was achieved. The measurement invariance test of the scale error learning orientation, with the three items L-4, L-5 and L-6, showed non-significant Δ χ^2^, therefore, it can be assumed according to Bialosiewicz et al. (2013) and Kleinke et al. (2017) that the error learning orientation has the same meaning over time. Consequently, there is no measurement variance.

Table 1: Series of CFA models investigating the measurement invariance between the two time points of error learning orientation

| Parameters constrained to be equal | χ^2^ | df | CFI | TLI | RMSEA | Δ χ^2^ | ΔCFI |
| --- | --- | --- | --- | --- | --- | --- | --- |
| 1. Unconstrained (configural invariance) | 32.327 | 13 | 0.951 | 0.921 | 0.074 | - | - |
| 2. Factor loadings (metric invariance) | 36.922 | 15 | 0.945 | 0.923 | 0.074 | n.s. | 0.006 |
| 3. Intercepts (scalar invariance) | 41.028 | 17 | 0.939 | 0.925 | 0.073 | n.s. | 0.006 |

Note: CFA = confirmatory factor analyses; CFI = comparative fit index; TLI = Tucker–Lewis index; RMSEA = root mean square of approximation; model comparison with Chi-squared test. **sign., p <.01.

**A4 Table: LGC models for enjoyment, anxiety and boredom over time, with gender, age, intervention group and error learning orientation as covariates**

|  | *Enjoyment* | | *Anxiety* | | *Boredom* | |
| --- | --- | --- | --- | --- | --- | --- |
|  | *Estimates* | *SE* | *Estimates* | *SE* | *Estimates* | *SE* |
| *Means* |  |  |  |  |  |  |
| Intercept | 2.29* | 1.02 | 1.52 | .78 | 5.69*** | 1.15 |
| Slope | .54 | .31 | .20 | .29 | -.28 | .44 |
| *Variances* |  |  |  |  |  |  |
| Intercept | .84 | .13 | .70 | .11 | 1.28 | .19 |
| Slope | .03 | .02 | .05 | .02 | .12 | .04 |
| *Covariance* |  |  |  |  |  |  |
| Slope with intercept | -.42 | 1.32 | -.58*** | .17 | -.52*** | .14 |
| *Predictors of emotions* |  |  |  |  |  |  |
| Error learning orientation T1 → intercept | .36*** | .08 | -.08 | .09 | -.29*** | .09 |
| Error learning orientation T1 → slope | .01 | .19 | -.11 | .12 | .02 | .13 |
| Gender (female) → intercept | -.43*** | .06 | .19** | .07 | .33*** | .07 |
| Gender (female) → slope | .37 | .28 | -.07 | .11 | -.13 | .10 |
| Age → intercept | .09 | .07 | .05 | .08 | -.16* | .07 |
| Age → slope | -.17 | .19 | -.10 | .11 | .03 | .12 |
| Intervention group → intercept | .12 | .07 | -.15* | .07 | -.02 | .07 |
| Intervention group → slope | -.18 | .22 | .01 | .11 | .02 | .10 |
| *Outcomes of emotions* |  |  |  |  |  |  |
| Intercept → error learning orientation T2 | .03 | .22 | -.05 | .14 | -.04 | .15 |
| Slope → error learning orientation T2 | .03 | .38 | -.11 | .17 | .24 | .18 |
| *Covariates predicting error learning orientation* |  |  |  |  |  |  |
| Intervention group → error learning orientation T1 | .07 | .07 | .07 | .07 | .07 | .07 |
| Gender (female) → error learning orientation T1 | .11 | .07 | .11 | .07 | .11 | .07 |
| Age → error learning orientation T1 | .21*** | .06 | .20*** | .06 | .20*** | .06 |
| Intervention group → error learning orientation T2 | -.01 | .13 | -.02 | .07 | -.02 | .07 |
| Gender (female) → error learning orientation T2 | .02 | .18 | .01 | .07 | .06 | .10 |
| Age → error learning orientation T2 | -.14 | .12 | -.15* | .07 | -.15 | .10 |
| Error learning orientation T1 → error learning orientation T2 | .75*** | .16 | .74*** | .09 | .75*** | .10 |

Note: Standardized estimates, ****p* < 0.001, ***p* < 0.01, **p* < 0.05; T1 = pre-test, T2 = post-test
